# Foreign Body Reaction (Immune Response) for Artificial Implants Can Be Avoided: An Example of Polyurethane in Mice for 1 Week

**DOI:** 10.3390/jfb14080432

**Published:** 2023-08-17

**Authors:** Irina Kondyurina, Alexey Kondyurin

**Affiliations:** 1 School of Medicine, University of Sydney, Sydney, NSW 2006, Australia; i.kondyurina@gmail.com; 2 School of Physics, University of Sydney, Sydney, NSW 2006, Australia; 3 Ewingar Scientific, Ewingar, NSW 2469, Australia

**Keywords:** foreign body reaction, implant, histology, polyurethane

## Abstract

Despite great success with artificial implants for the human body, modern implants cannot solve major health problems. The reason is an immune reaction of organisms to artificial implants, known as the foreign body reaction. We have found a way to avoid or decrease the foreign body reaction. The surface of an artificial implant is modified with condensed aromatic structures containing free radicals, which provide a covalent attachment of host proteins in a native conformation. The total protein coverage prevents the direct contact of immune cells with the implant surface, and the immune cells are not activated. As a result, the immune response of the organism is not generated, and the artificial implant is not isolated from the tissue; there is no collagen capsule, low activity of macrophages, low cell proliferation, and low inflammatory activity.

## 1. Introduction

Modern artificial implants have successfully worked in organisms for more than 20 years. It was estimated that, only in the USA, 20 million people have had biomedical implants to save their lives [1] The success of the medical implant industry and surgery practice has been proven by the high number of successful operations. However, artificial implants are not a complete solution. Research has shown that 73% of people who received artificial heart implants survived after 9 years and 65% survived after 18 years. In the case of artificial aorta implants, 85% of people survive after 5 years. 

The problem is a full integration of the implant into an organism. An intrusion of any artificial material into an organism causes a reaction of the organism’s immune system called the foreign body reaction or foreign body response (FBR) [2]. The immune reaction on the foreign body protects the organism against bacteria, viruses and injuries and causes an isolation of the implant from the organism tissue that can break the functionality of the implant. In the worst-case scenario, the implant must be removed or replaced. This involves a secondary operation that increases the risk of lethality significantly, especially for elderly people, who mostly need the implants. Research has shown that 100% of all artificial implants cause an immune reaction, and 35% of them require a secondary operation. 

The immune response to an artificial implant is manageable with immunodepressant therapy [1,3,4]. The immune system of an entire organism is depressed to decrease or to avoid foreign body reactions to the implant. Such therapy is a common way to avoid implant isolation. However, such a depressed immune system cannot protect an organism against any disease. Most of such patient deaths are caused by another disease, not connected with the implant itself. 

**Figure 1:**
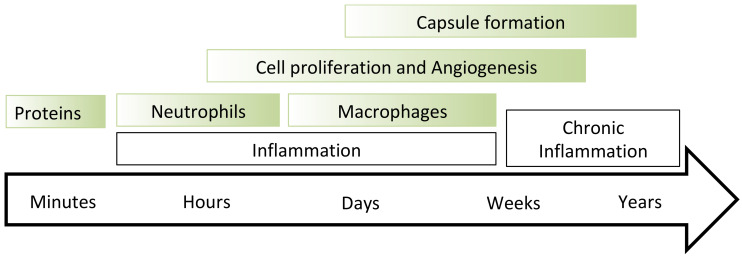
Scheme of the foreign body reaction with the main stages from the time of first contact of the implant with the organism tissue. The scheme is adapted from the literature data [5] for untreated polyurethane.

The foreign body reaction is ignited by the implant’s surface interaction with the host’s proteins (Figure 1) [5]. The protein molecules are adsorbed on the implant surface, and the initial conformation of the protein is changed [6]. The immune cells recognise the protein with a changed conformation as a foreign protein, and immune cells are activated to destroy the protein. The cycle of attachment—changed conformation—destruction is repeated and repeated until the immune cells activate the next level of the immune response, such as neutrophil and macrophage collection near the implant surface, intensive cell proliferation and angiogenesis, collagen encapsulation and calcification (in the worst case) of the collagen capsule to isolate completely the implant [6,7]. The foreign body reaction is detected by immune cell activity such as neutrophils and macrophages, intensive cell proliferation, inflammatory reaction, specific cytokines and a collagen capsule surrounding the implant. 

A decrease in the foreign body reaction was observed earlier in our studies on ion beam-implanted polyurethane implants in mice and rats [8]. The collagen capsule was not formed near the carbonised polyurethane surface even after 5 months in the mice organisms. The low immune response was based on the protein covalent attachment on the carbonised surface due to the presence of free radicals at the edges of grapheme sheets [9,10]. However, a detailed histological investigation of the tissue surrounding the implant was not performed. 

In the present study, we investigated the immune response to polyurethane implants treated by plasma immersion ion implantation as a kind of general ion beam implantation method and described a method to avoid the foreign body reaction for polyurethane implants without immunodepressant therapy. 

## 2. Materials and Methods

**Figure 2:**
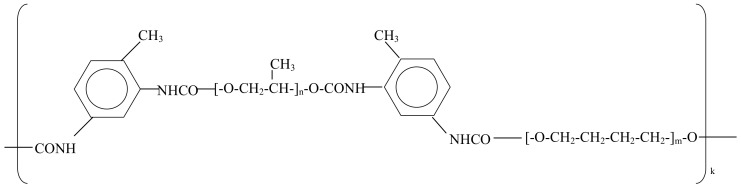
The general formula of PPG-DI-PTHF polyurethane. Crosslinking bonds are not shown.

Polyurethane (Figure 2) was synthesised from polypropylene glycol terminated by toluenediisocyanate (Aldrich, Macquarie Park, Australia) and polyoxitetramethylene (Aldrich) with a deficit of hydroxyl groups that gave 3D crosslinking of the macromolecules. The details of the synthesis were described elsewhere [11]. The synthesised polyurethane was swollen in heptane to remove all non-crosslinked molecules and then air-dried to the complete evaporation of heptane controlled by FTIR spectra. The final films of 0.3 mm thickness and 150 mm in diameter were used. 

The polyurethane films were treated by plasma immersion ion implantation (PIII) by 20 keV energy nitrogen ions of 40, 80, 200, 400 and 800 s in treatment time. The details of the PIII treatment were described elsewhere [12]. The next day, the films were cut into 3.5 diameter disks and implanted subcutaneously in the mice. This experiment was approved by the University of Sydney Animal Ethics Committee (protocol number K20/12-2011/3/5634) and conducted in accordance with the Australian Code of Practice for the Care and Use of Animals for Scientific Purpose. After 7 days, the implants were taken from the mice; sliced with microtome and investigated with Milligan’s trichrome, hematoxylin–eosin, F4-80, Ki-67 and Von Willebrand factor staining following the standard protocols [13]. The micro-photos were completed with a Nikon microscope (Japan). The objectives ×4 for low-resolution images and ×20 for high-resolution images were used. 

An analysis of protein attachment on a polyurethane surface was completed with bovine serum albumin (BSA) as an example. The polyurethane samples were incubated in BSA solution for 24 h at room temperature. The protein was diluted to 20 μg/mL in 10 mM sodium phosphate buffer at pH 7. After incubation, the samples were regressively washed in the buffer and in deionised water. For the covalently attached protein analysis, the polyurethane samples were washed in 2% sodium dodecyl sulphate (SDS) solution in deionised water at 70 °C for 1 h. 

FTIR ATR spectra in a range of 400–4000 cm^−1^ were recorded with the spectrometers Nicolet Magna 650 (USA), Digilab FTS (USA) and Excalibur (USA). ATR spectra were recorded with ATR crystal Ge, the angle of the beam incident was 45 degrees and number of scans was 100. The spectral resolution was 4 cm^−1^. The contact angle of deionised water and diiodomethane (Aldrich) on the polyurethane surface was measured with a DS-10 device (KRUSS, Hamburg, Germany). Micro-Raman spectra were recorded in 180° geometry excited by the Nd:YAG laser line of secondary harmonics (532.14 nm wavelength) on a diffraction double monochromator HR800 (Jobin Yvon). The electron spin resonance spectra (ESR) were recorded using an Adani electron spin resonance spectrometer (Adani, Minsk, Belarus). The surface topography of the polyurethane was measured with atomic force microscopy (AFM) using a Park System device (Park System, Suwon, Republic of Korea) in tapping mode with a speed of 1 Hz and amplitude 10 nm. Micro-photos of the polyurethane surface were completed using an optical microscope MBS-10 with an attached video camera. 

## 3. Results 

### 3.1. Analysis of PIII-Treated Polyurethane Surface

The untreated polyurethane surface is hydrophobic, with a water contact angle of 93 degrees. The water dropping measurements show a significant decrease in the contact angle to 35–45 degrees in the 15 min after the PIII treatment. The contact angle increases with the storage time after the PIII treatment up to 60–70 degrees. The angle is stabilised a week after the PIII treatment. These changes were observed to be in a similar statistical range for all PIII treatment times from 40 s to 800 s. The contact angles of the water and diiodomethane were used for the calculation of the surface energy and its parts. The surface energy is an energetic parameter of the surface to describe an interaction with the adsorbed molecules. The surface energy increased from 33 mN/m for the untreated polyurethane to 65–67 mN/m for the freshly treated polyurethane. A week after the PIII treatment, the surface energy decreased, with stabilisation at 45–50 mN/m. The stabilised level of the surface energy was much higher than the surface energy of the untreated polyurethane. The dispersal part of the surface energy increased from 31 mN/m for the untreated polyurethane to 40–44 mN/m for the freshly treated polyurethane and decreased with a storage time up to 37–39 mN/m. The polar energy changed more dramatically. The polar part calculations showed a value of 2 mN/m for the untreated polyurethane. A short time after the PIII treatment, the polar part of the surface energy increased to 17–27 mN/m and then decreased and was stabilised at 8–14 mN/m. Therefore, the surface was hydrophilic after the PIII treatment. 

**Figure 3:**
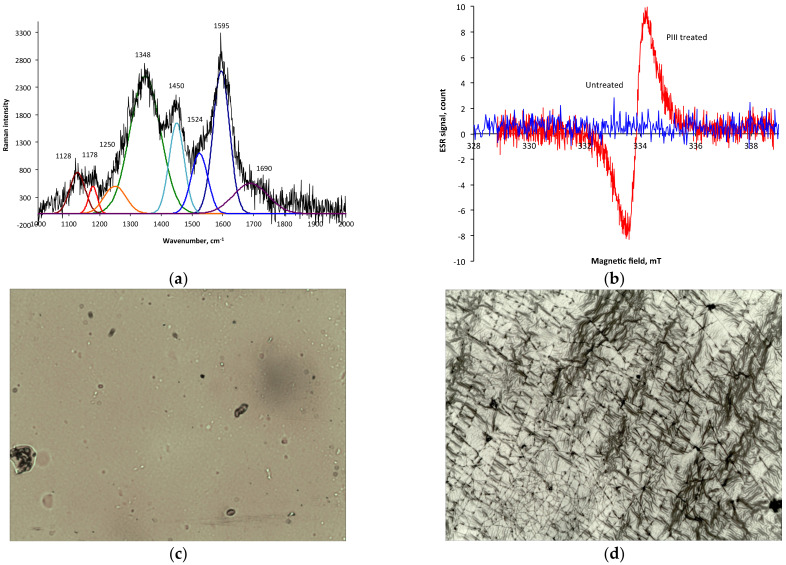

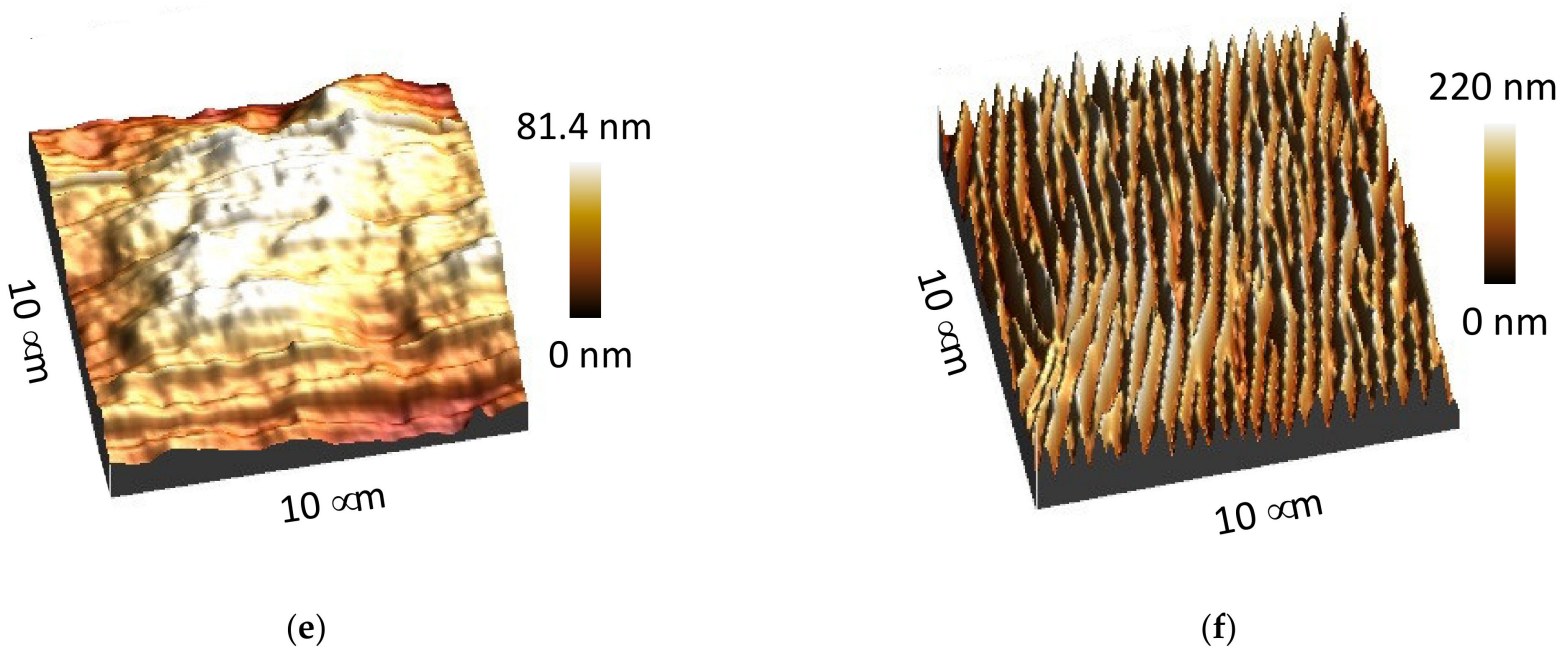
(**a**) Micro-Raman spectra of 20 keV nitrogen ion PIII-treated polyurethane at 800 s (bottom spectrum) fitted with Gauss functions (colour lines). Two new strong peaks at 1348 and 1595 cm^−1^ show the presence of graphite planes with defects in the PIII-treated polyurethane. (**b**) Electron spin resonance spectra of untreated polyurethane (blue) and PIII-treated polyurethane (red) recorded from the samples after 3 months of storage in the laboratory. Optical micro-photos of polyurethane untreated (**c**) and after 800 s of PIII treatment by 20 keV energy nitrogen ions (**d**). The PU was synthesised and treated on silicon wafer to exclude any deformations after the treatment. Size of the images is 0.24 × 0.19 mm^2^. A developed surface structure with waves and cracks is observed after the PIII treatment. AFM 3D 10 × 10 μm^2^ area image of the polyurethane surface untreated (**e**) and treated with PIII for 40 s with 20 keV nitrogen ions (**f**).

The Raman spectrum of the PIII-treated polyurethane (Figure 3a) contained new strong lines at 1348 and 1595 cm^−1^. These two lines were interpreted as resonance Raman lines of the carbonised top layer: the D and G peaks. The G peak is E_2g_ vibrations in a graphitic structure with sp^2^ hybridisation of the valence electrons. The D peak is A_1g_ vibrations in a graphitic structure. The ratio I(D)/I(G) = 1.77 and G peak position corresponds to the I area in a Robertson/Ferrari diagram [14] that shows a nanocrystalline graphite with a characteristic size of graphitic islands of L_a_ = 2.5 nm separated by amorphous carbon with sp^3^ hybridisation. 

A signal of unpaired electrons in the modified polyurethane was detected with the electron spin resonance spectra. The electron spin resonance spectra of the untreated polyurethane and the PIII-treated polyurethane at 800 s by nitrogen ions with 20 keV energy are presented in Figure 3b. The untreated polyurethane film did not have a signal higher than the noise level. The PIII-treated film had a strong signal with a g factor of about 2.0028. The signal of the g factor corresponds to unpaired electrons of carbon-free radicals at the edges of aromatic structures in graphite- or graphene-like structures [15]. The spectra showed that the free radicals in the modified layer appeared after the PIII treatment and then remained when it was stored for a further 3 months. With such a long shelf-life, free radicals are stabilised by π-electrons of the aromatic rings of graphite-like structures. 

The surface topography of PPG-DI-PTHF-0.35 polyurethane was changed dramatically after the PIII treatment and was visible with an optical microscope and atomic force microscope (Figure 3c–f). The treated surface was wrinkled and cracked. The directions of the wave and cracks were random. The surface topography remained stable after a month-long storage time. The topography viewed was similar to polyurethane free-standing films and polyurethane coating on a hard substrate (silicon wafer). Therefore, the surface wrinkles and cracks were not the result of deformation of the sample. The surface topography corresponded to a hard surface layer with internal stresses over the soft bulk layer. 

**Figure 4:**
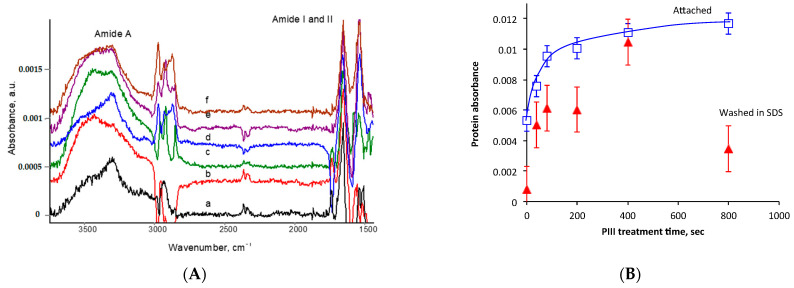
(**A**) FTIR ATR spectra of attached BSA on the untreated and PIII-treated polyurethane of PPG-DI PTHF with a 0.35 NCO/OH ratio. From bottom to top: (a) untreated polyurethane and (b) 40, (c) 80, (d) 200, (e) 400 and (f) 800 s PIII treatment times with 20 keV nitrogen ions. The spectra of the corresponding polyurethanes are subtracted. (**B**) FTIR ATR spectra absorbance of the Amide I line of BSA attached to the polyurethane of PPG-DI PTHF with a 0.35 NCO/OH ratio with the PIII treatment time: blue is the attached BSA, and red is BSA attached and then washed off in SDS for 1 h at 70 °C. The absorbance of Amide I of the protein was normalised for the absorbance of the 1100 cm^−1^ line related to vibrations in polyurethane.

Bovine serum albumin (BSA) was used as an example in the attachment experiment. The polyurethane samples were incubated overnight in BSA solution in a PBS buffer. The control samples were incubated in buffer solutions only. These were prepared the same way and at the same time as the protein-attached samples. Then, all samples were rinsed in deionised water and dried overnight, and the FTIR ATR spectra were recorded. The spectral lines of the protein were not visible in row of spectra of the samples as recorded due to the high intensity of the polyurethane lines in comparison to the protein lines. The low intensity of the protein lines corresponded to a very thin BSA protein monolayer (about 5 nm) in comparison to the high-intensity polyurethane spectrum. The spectra of the control samples were subtracted from the spectra of the protein samples treated at the same PIII treatment time with an adjusted subtraction factor to minimise the absorbance of the polyurethane lines (Figure 4A). The subtracted spectra showed the Amide A line at 3300 cm^−1^, the Amide I line at 1650 cm^−1^ and the Amide II line at 1540 cm^−1^. The line positions of Amide A and Amide II in the spectra of pure polyurethane and pure protein were similar. These lines in the subtracted spectra could be interpreted as related to both polyurethane and protein. Therefore, these lines could not be used for analysis of the protein attachment. The Amide I line in the spectra of pure polyurethane was observed at 1720 cm^−1^, and the Amide I line in the spectra of pure protein was observed at 1650 cm^−1^. Due to these different positions, the Amide I line could be used for analysis of the protein attachment. The absorbance of the Amide I line normalised on the line of polyurethane vibrations at 1100 cm^−1^ as a stable intensive line is presented in Figure 4B according to the PIII treatment time. The amount of attached BSA increased with the PIII treatment time up to the saturation level. The saturation level observed after 800 s of PIII treatment time was double that for the untreated polyurethane. 

The detergent SDS was used for washing the noncovalently attached BSA molecules from the polyurethane surface. The experiment was completed with the same samples of polyurethane after FTIR spectra recording. After washing in the detergent, all samples were rinsed in deionised water and dried overnight. The FTIR ATR spectra of the control samples were subtracted from the spectra of the protein samples, as described above, and absorbance of the protein Amide I line was calculated and presented in Figure 4B. The protein lines in the spectrum of the untreated polyurethane were not observed and were under the noise level of the spectrum. The spectra of the SDS-washed samples showed the Amide I line of the attached protein only for the PIII-treated polyurethane. Therefore, the protein was almost washed from the untreated polyurethane. It showed that the protein on untreated polyurethane attached due to physical forces was disturbed by the physical forces of the detergent’s molecules. Washing in the detergent was sufficient to remove whole protein layers attached to the untreated surface due to physical forces. 

The amount of attached protein on the PIII-treated polyurethane after washing in the detergent solution was lower than the amount of attached protein before washing. However, a significant portion of the attached protein remained on the surface after washing, despite the detergent’s physical forces. This showed that this protein was attached chemically to the surface. The ratio of attached-to-remained after detergent washing varied from 30% for the 800 s PIII-treated sample to 95% for the 400 s PIII-treated sample, with average amount of 63% for all PIII-treated samples.

### 3.2. Histology Analysis of the Tissue around the Polyurethane Implant

**Figure 5:**
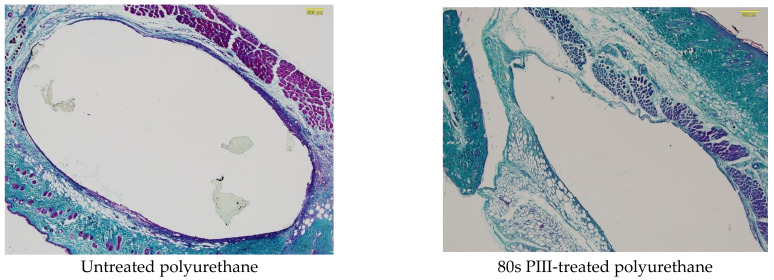
Images of the histological samples of polyurethanes stained with Milligan. The size of the images is 3.45 × 2.6 mm^2^. The implant position is in the centre of the ovals. Usually, the polyurethane sample is removed during microtome cutting, but sometimes, small pieces of the polyurethane samples remain.

The histological images of the sections stained with Milligan’s trichrome showed that a collagen capsule was formed around all samples of the polyurethane implants (Figure 5). The polyurethane implants were removed during microtome cutting due to their very elastic polyurethane properties in comparison with the surrounding tissues. In all the images, polyurethane was in the centre of the empty ovals. In some cases, the residuals of the polyurethane implants remained in the sections and stained slightly. The capsule continuously covered the implants, isolating the implant from the tissue. The observed ruptures in some capsules were due to microtome cutting, as shown by the character of the ruptures. The capsule for the untreated polyurethane implants had more intensive colouring and was broader than for the PIII-modified implants. However, the capsule thickness appeared to be independent of what surrounded the implants, whether muscle or connectivity tissues. 

**Figure 6:**
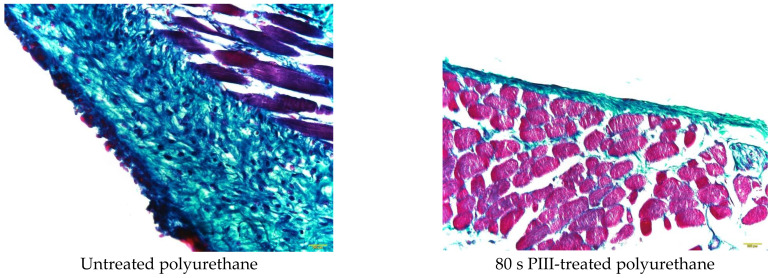
Microphotographs of the histological tissue samples stained with Milligan for untreated and PIII-treated polyurethane. The empty space on the figures is the implant position. The size of the images is 0.69 × 0.52 mm^2^.

The thickness of the capsule was analysed quantitatively using high-resolution images of the tissue near the implants. Representative examples of the section stained with Milligan’s trichrome in high resolution for the polyurethane implants with different PIII treatment times are presented in Figure 6. 

The collagen shell stained with Milligan’s trichrome was well developed in the tissue surrounding the untreated polyurethane implants. The collagen fibres were structured and directed parallel to the implant surface at a distance of 40–60 μm. This part of the shell was characterised by a dense collagen structure. The collagen fibres were disordered at a distance of 60–120 μm from the implant surface. This part of the shell was less dense but denser than the collagen and elastin fibre structures in the normal tissue. In some samples, a very dense collagen structure was observed in the first approximately 10 μm layer from the implant surface.

The PIII treatment of the polyurethane implant changed the collagen shell in the organism (Figure 6). A dense collagen shell was not observed. A collagen shell near the implant surface was not developed, and the collagen fibres were not directed but were disordered and rare. The whole collagen shell was thinner than for the untreated polyurethane implant. For some samples, the shell was observed in one to two cell layers between the implant surface and the muscle cells. In some samples, a dense collagen shell was observed in the thin surface layer (up to about 10 μm), which was in closest contact with the implant. All these differences in the collagen shell were observed in different animals and were not attributed to a specific immune reaction of one mouse. 

The average thickness of the collagen shell was calculated and is presented in Table 1. The staining of all the slides was completed in one day, the microphotographs were completed using one microscope in one day with adjusted intensity and optical elements of the microscope and the analysis of the images was completed for all microphotographs simultaneously. This allowed the results between the different samples in the whole batch to be compared quantitatively. 

The results showed that the average thickness of the collagen capsule in all PIII-treated samples was significantly less for the PIII-treated polyurethane implants (41 μm) than for the untreated implants (102 μm) (*p* < 0.001). A significant decrease in the thickness from 102 μm to 46 μm was observed after a short 40 s PIII treatment time (*p* < 0.01). This difference between the untreated and PIII-treated samples increased with the PIII treatment time. The thinnest thickness (38 μm) was observed for the 200 s PIII treatment time.

**Figure 7:**
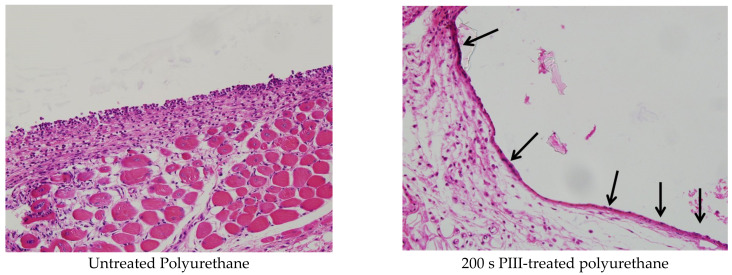
Microphotographs of the histological tissue samples stained with hematoxylin–eosin for the untreated and 200 s PIII-treated polyurethane implants. The empty space in the figures is the implant position. Positions of the macrophages are shown with arrows. The size of the images is 0.69 × 0.52 mm^2^.

Microphotographs of the H&E-stained tissue are shown in Figure 7. The tissue near the untreated polyurethane implant contained macrophages, monocytes and fibroblasts. Multinuclear foreign body giant cells were not observed. The macrophages and monocytes were mostly distributed near the surface of the implant in the capsule. Their concentrations were highest in the capsule near the implant surface. The fibroblasts were positioned mostly at a distance from the implant surface, where the collagen fibres dominated. There was no cell lysis or necrosis.

The tissue near the PIII-treated implant was less disturbed (Figure 7). Most cells in the capsule were fibroblasts, and a few cells were found in other spots, like macrophages and monocytes (marked with arrows). Macrophages and monocytes were predominantly in the contact zone of the implant surface. In some images, it was clear that the spots with macrophages and monocytes were distributed along the implant surface at the same periodicity as the cracks and waves of the polyurethane surface. Such cell distribution was observed for all PIII treatment times. Similar capsule images were observed in cases when the modified implant was in contact with the connective tissue or muscle tissue or adipocytes cells. In some places where the tissue contacted the modified implant, the capsule was not observed at all. The cells, which were in contact with the implant surface, were fibroblasts or connective tissue fibres.

**Figure 8:**
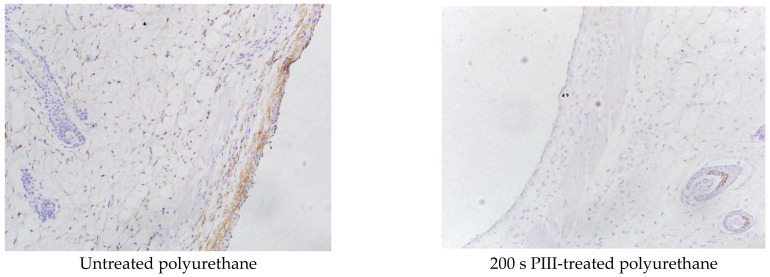
Microphotographs of the histological tissue samples stained with F4-80 antibody for the PPG-DI-PTHF-0.35 polyurethane implants. The empty space in the figures is the implant position. The size of the images is 0.69 × 0.52 mm^2^.

Staining with the F4-80 antibody was used to detect the presence of macrophages in the tissue surrounding the polyurethane implant. A high macrophage activity was observed in the collagen capsule near the surface of the untreated polyurethane implant (Figure 8). 

The rest of the tissue outside of the collagen capsule showed low macrophage activity. The same low macrophage activity was observed in the tissue surrounding the PIII-treated implant (Figure 8). For some samples, macrophage activity near the implant surface was not observed. 

The integral area of the tissue with brown colour and the integral intensity of the colour were analysed to estimate the macrophage activity in the tissue. The results of macrophage activity dependent on the PIII treatment time are presented in Table 2. The highest macrophage activity was observed in the tissue surrounding the untreated polyurethane implant and 800 s PIII-treated sample. The lowest macrophage activity was observed for the 200 s PIII-treated implants. The difference between the untreated and 200 s PIII-treated samples was significant (*p* < 0.001). 

**Figure 9:**
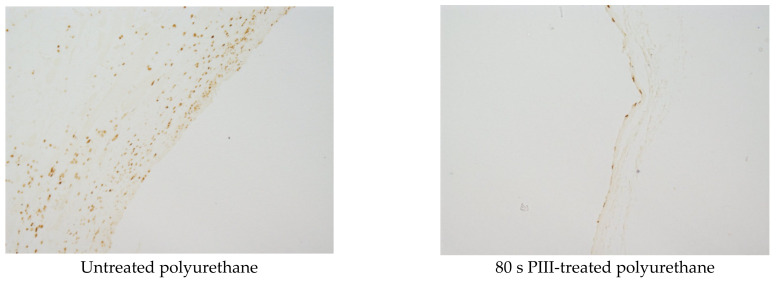
Microphotographs of the histological tissue samples stained with Ki-67 antibody for untreated and 80 s PIII-treated polyurethane. The empty space in the figures is the implant position. The size of the images is 0.69 × 0.52 mm^2^.

The Ki-67 antibody was used for detection of the cell proliferation activity in the tissue surrounding the polyurethane implants. An example of characteristic images for untreated and PIII-treated implants with different PIII treatment times are presented in Figure 9. The colour distribution and intensity show a high cell proliferation activity in the tissue near the untreated polyurethane implants. The maximum activity is observed in the collagen capsule, and the activity gradually decreases with distance from the implant. 

The cell proliferation activity near the PIII-treated implants was much less (Figure 8). In some samples, the activity was observed only in a thin surface layer corresponding to one cell thickness. The most intensive activity was observed in the areas of new vessel formation. 

For a quantitative analysis of the cell proliferation activity, the colour area and density of Ki-67 staining were analysed and are presented in Table 3. The activity decreased with the PIII treatment time, and the minimal value was observed at 400 s of the treatment time (*p* < 0.001). This trend was observed in the area and the density of stained cells.

**Figure 10:**
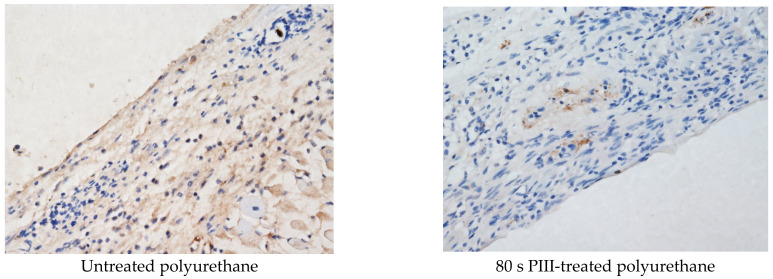
Microphotographs of the histological tissue samples stained with the von Willebrand factor for untreated and 80 s PIII-treated polyurethane. The empty space in the figures is the implant position. The size of the images is 0.69 × 0.52 mm^2^.

The von Willebrand factor (vWF), as a proinflammatory protein and a key player in haemostasis, was analysed in the tissue surrounding the polyurethane implants. The microphotographs of the tissue stained with the vWF antibody are presented in Figure 10. The tissues near the untreated polyurethane implants accumulated a large amount of the vWF. Some samples showed the highest concentration of the vWF in the capsule, but some samples showed a distribution of the vWF in the whole tissue far from the capsule (an example is in Figure 10). A high vWF amount was also observed in the new vessels. In the PIII-treated samples, the vWF was concentrated in spots outside the capsule. A much lower amount of vWF was observed in the tissue surrounding the PIII-treated polyurethane implants. In some samples, a coloured area was not observed at all. In all these samples, the capsule did not contain vWF. 

For quantitative measurements, the area of vWF-stained tissue was calculated (Table 4). The measurements showed that the tissue near the implants in the 80–800 s range of the PIII treatment time had a significantly lower area of vWF than the tissue surrounding the untreated and 40 s PIII-treated implants (*p* < 0.001). The difference between the tissues near the PIII-treated implants in the 80–800 s range was not significant (*p* > 0.05). 

## 4. Discussion

The foreign body reaction (FBR) is a key problem for modern artificial implants [6]. It does not only limit implant development, but it limits the number of patients to whom implants can be applied. An absence or reduction in the FBR would significantly improve implants’ capacity to save lives. However, despite how all modern implants satisfy the physical and chemical stability, non-immunogenicity and nontoxicity requirements [16], all these implants still cause an immune response in an organism. This is why there is a widely held opinion that FBR cannot be avoided [5]: “All materials implanted into humans and animals elicit ‘foreign body’ interactions, surrounding the materials with a protective capsule.”

Attempts to avoid or decrease the immune reaction to artificial implants can be found in the literature [17,18,19,20,21]. All these attempts were based on stopping the immune reaction at some stage. For example, systemic corticosteroids decrease immune function and fibrosis—in particular, they decrease the number of myofibroblasts [22]. However, our knowledge of the mechanism of the immune reaction is limited. The immune reaction is complicated and self-regulated, with multiple ways and steps. Blocking one step in the immune response will not prevent a protective capsule formation.

Therefore, the most effective way to avoid the foreign body reaction is to not ignite it. This way is used in the present study.

The untreated polyurethane implant shows a minor foreign body reaction with the formation of the capsule filled with macrophages and fibroblasts. Giant cells or necrosis were not observed in any samples. The thickness of the capsule of about 100 μm surrounding the implant corresponds to the literature data for biocompatible implants used in medical practice [4,23]. Therefore, the untreated polyurethane corresponds to the biocompatible materials following the organism’s reaction.

The organism’s response to PIII-treated polyurethane was significantly reduced, showing that the average thickness of the capsule was significantly less near the treated polyurethane than near the untreated polyurethane. In some cases, the capsule was not formed near the PIII-treated polyurethane surface at all. In addition, separate macrophages were observed on the surface, with large distances between them, whereas, in the other cases, only normal tissue was observed. In some cases, only one irregular layer of fibroblasts with thin, nonstructured collagen fibres was observed.

Acute inflammation and capsule formation are usually observed 5–7 days after surgery. Chronic inflammation is observed following this period, with a formation of a thick, isolating capsule around the implant. However, if the capsule around PIII-treated polyurethane is not observed 7 days after the operation, it is possible that the capsule might not be formed later, because a delayed formation of the capsule is less likely. Therefore, there are cases when the capsule is not formed or has significantly less thickness with a significantly lower number of immune cells. 

For discussion of the histochemistry results, this study considered a general scheme of the immune reaction on an implant (Figure 1) [5] where the reaction was characterised by stages of the organism response, which became more complicated and intensive with time until the total isolation of the implant from the body. 

**Figure 11:**
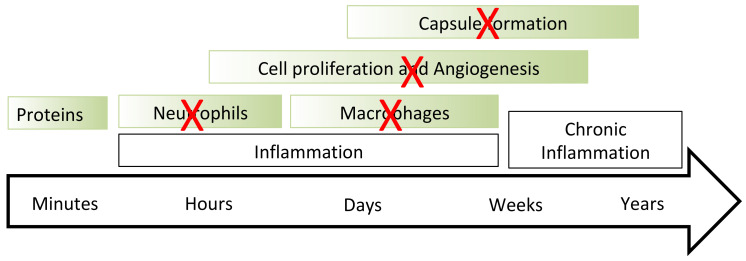
Scheme of the foreign body reaction with the main stages from the time of first contact of the implant with the organism tissue. The scheme is adapted from the literature data [5] for PIII-treated polyurethane. Red crosses show low or absent activity observed in the experiments with PIII-treated implants.

The histochemistry results showed the significant differences in the immune responses of the mice organisms with untreated and PIII-treated polyurethane implants (Figure 11). At first, the von Willebrand factor concentration in the tissue near the PIII-treated implants differed from that near the untreated implants. The literature [24] has demonstrated that the von Willebrand factor is closely related to the recruitment of neutrophils in the injured tissue near the implant. When the von Willebrand factor concentration increases, neutrophils are collected in the injured tissue and activated. The images of the von Willebrand factor assay in the tissue near the untreated polyurethane implant corresponded to a moderate immune reaction on the implants, as seen in the literature [25,26]. The lower von Willebrand factor concentration in the tissue near the PIII-treated implant meant that there was a less intensive inflammation and lower concentration and activity of neutrophils. Therefore, the second stage had a lower intensity for the PIII-treated implant than for the untreated implant. 

Macrophages are recruited at the next stage of the immune reaction. The role of macrophages is to remove the foreign body and/or to utilise cell apoptosis products. Macrophages are recruited and activated via the expression of cytokines released by neutrophils when the foreign body cannot be destroyed in the body. The concentration and activity of macrophages near the untreated implant show a moderate reaction of the organism consistent with the literature data [6,27,28,29]. The lower concentration and activity of macrophages in the tissue near the PIII-treated implant means that the macrophages are recruited and activated with much fewer signals from neutrophils. Therefore, the third stage of the immune response has a lower intensity. 

The new vessel formation and cell proliferation at the next stage provide a transport of immune cells to the injured tissue. These processes are ignited by released cytokines from the immune cells, which cannot exclude the foreign body. At this stage, the immune response includes deep transformation of surrounding tissues, which become involved in the healing process. The organism prepares to intensify the immune attack. The cell proliferation process in the tissue surrounding the untreated implant is much more intensive than in the tissue far away from the implant. The intensity at this stage is consistent with the literature on other implanted materials [6,27,28,29]. However, the cell proliferation activity in the tissue surrounding the PIII-treated implant is much less. Cell proliferation is not detected, suggesting that the organism does not recognise the implant as a foreign body. 

With time, the implant is encapsulated with collagen fibres. The fibroblasts are activated and build a crosslinked collagen capsule around the implant. At this stage, the organism tries to isolate the foreign body from the tissues. The immune response is transformed to chronic inflammation. The thickness of the capsule depends on the intensity of the foreign body reaction. The capsule around the untreated polyurethane implant is about 100 μm, which corresponds to a moderate immune reaction for most polymer implants [4,23]. 

The capsule surrounding the PIII-treated implants is much thinner. This collagen capsule consists of rare disordered fibres, whereas a capsule of dense ordered collagen fibres surrounds the untreated implant. In some cases, the capsule near the PIII-treated implant is not observed at all. Therefore, the host does not recognise the PIII-treated implant as a foreign body that must be isolated. 

All these observations are related to acute inflammation observed in the 7 days after the operation. According to the literature [4,23], the capsule thickness for untreated implants grown after the acute stage increases during the chronic stage. In addition, previous long-term investigations with ion beam-treated hard polyurethane have shown the absence of an immune response in the organism after 5–6 months. Because of this absence in these long-term experiments with the new polyurethane, we predict that the intensive chronic inflammatory reaction to PIII-treated polyurethane is unlikely due to the low inflammatory reaction during the acute stage. Therefore, low and absent foreign body reactions are achievable. 

A thin capsule on the ion beam-treated implant is observed from the implant side [8]. The collagen capsule is well attached to the untreated polyurethane implant. However, the capsule is not found on ion beam-implanted polyurethane surfaces with a carbonised top surface. The collagen structures are observed in the bottom of the crack of the carbonised layer, where the patches of untreated polyurethane are accessible by host proteins, phagocytes and fibroblasts. 

A similar weak immune reaction was observed for ion beam-treated polyurethanes in the experiments of Suzuki and colleagues [30]. Polyurethane treated by Ar+ were implanted into a rabbit for 16 days, and a weak intensity of the inflammatory reaction was observed. A similar effect of biocompatibility of the polymer materials after the ion beam treatment was observed during in vivo experiments with ePTFE grafts. An ePTFE graft was treated by an ion beam and implanted in dogs’ femoral arteries. The control sample showed thrombosis after 3 days, whereas the modified sample was clear after 180 days [31]. However, the mechanism responsible for the weak immune reaction was not discovered at that time. 

In the present study, the attempt to prevent phagocyte activation is based on the knowledge of specific adsorption of the host proteins on the PIII-treated implant surface. 

It is clear now that the immune response of an organism starts from a specific adsorption of host proteins on the surface of the artificial implant [32]. Then, the phagocytes recognise the adsorbed proteins on the implant surface and become activated. The activated phagocytes release specific cytokines, chemokines, interleukins and other factors, which ignite the next steps of the inflammation reaction. The activation of phagocytes is a complex process and includes local cytokine expression, transformation of the macrophages and giant cells. At some stage, the fibroblasts are activated, proliferated and ignited to produce collagen to form a capsule around the implant. 

The host proteins in organisms exist in a hydrophilic environment such as the cytoplasm, extracellular liquids, blood and lymph nodes. The biologically active conformation of the protein molecule is optimised for such an environment. When the host proteins come into contact with a surface of the artificial implant, the environment for the host proteins is changed: the protein is in contact with the implant, not with water molecules [33]. The protein molecule is adsorbed on the implant surface and changes the conformation due to different intermolecular interactions with the implant surface in comparison with the organism’s (hydrophilic) environment [28]. The conformation of the absorbed protein depends on the surface properties [34]. However, all artificial implants have a surface energy in the range of 20–45 MJ/m^2^, corresponding to the surface energy of the implant material [35]. This is much lower than the water surface energy of 72 MJ/m^2^. Therefore, the protein conformation changes to adjust the hydrophobic interactions with the implant. 

In contradiction with the untreated polyurethane, the PIII-treated polyurethane implant is highly hydrophilic. The contact angle of the carbonised surface is 35–45 degrees for freshly treated polyurethane. The surface energy of the treated polyurethane 10 min after the PIII treatment is 60–65 MJ/m^2^. Such a high surface energy and hydrophilicity is enough to absorb the water molecules and keep the hydrophilic intermolecular interactions with the proteins in such a way that the proteins are surrounded by water molecules and do not change the initial conformation at the point of absorption. This effect of hydrophilicity was observed for other polymers after the PIII, ion beam treatment and plasma treatment [36]. For example, the literature shows a surface energy of 71 MJ/m^2^ for freshly PIII-treated nylon [37] and 77.9 MJ/m^2^ for the freshly treated polyvinyl chloride (PVC) [38]. It maintains the conformation of the absorbed protein and its activity. For example, the conformation of horseradish peroxidase (HRP) attached to PIII-treated polyethylene remains unchanged or close to the biologically active conformation, as observed by the Amide I line in the FTIR ATR spectra [39]. The enzyme activity of HRP attached to PIII-treated Teflon remains high [40]. 

At the same time, the protein is attached covalently to the PIII-treated polyurethane implant. The covalent attachment is key to the strong anchoring of the protein to the implant surface. The protein attachment to the untreated implant can be provided with different kinds of intermolecular interactions. The van der Waals forces, dispersion interactions, dipole–dipole interactions, ionic interactions or hydrogen bonds can hold the protein on the surface. However, these interactions are flexible and can be easily replaced with water–protein interactions. The more hydrophilic the surface, the easier the protein can be washed with a detergent or just a buffer [41]. Such a weakness of the intermolecular interaction is the reason for the Vroman effect, where the proteins can be easily replaced with other proteins in protein mixture solutions or in an organism [33]. Such flexibility of the protein adsorption can lead to the unpredictable attachment of proteins, including the signal proteins for igniting an immune reaction.

The covalent attachment of a protein is available with artificial chemical linkers [42,43,44]. The linker molecule is designed in such a way that one reactive group fixes the linker molecule on the surface and the second reactive group anchors the protein molecule to the linker. However, the chemistry of linker molecules is complicated. In most cases, the linker molecules are toxic and cannot be used in an organism. Also, the effectiveness of the linkers to bind the protein is much less than the total coverage. In such cases, part of the surface is not covered, and the immune system can recognise the surface as a foreign body. 

The PIII-treated polyurethane surface can be covered completely. The protein monolayer forms total coverage (carpet) on the PIII-treated polymer surface [45], including polyurethane. The protein layer covalently attached to the PIII-treated polyurethane implant is not washable. The protein molecules covalently attached to the PIII-treated surface cannot be replaced with other proteins, and the Vroman effect is not observed [46]. 

The covalent attachment of a protein on PIII-treated polyurethane is provided via the reactions of free radicals, which are formed when the polyurethane macromolecule is destroyed under high energy ion bombardment, with the formation of condensed aromatic structures like graphene or graphite. The free radicals are observed in the polyurethane implant after a long period of storage. The free radicals on the edges of the graphitic planes can be stabilised by π-electrons of the aromatic structure [9,47] and remain active before the protein molecule approaches the modified surface. The literature shows that the reaction of free radicals appeared in polyethylene after ion beam treatment with amino acids [48]. When the free radicals react with the protein molecule, a covalent bond is formed [39]. As a result, the protein molecule bonds to the carbonised surface layer of the PIII-treated polyurethane implant. 

The molecules with free radicals in a solution are widely considered to accelerate the aging of an organism, because the host protein molecules can be damaged in free radical reactions [49]. The results of the protein damaging and oxidation can accelerate cell lysis and necrosis. These effects were not observed in the surrounding implant tissue. The effect of free radicals on the protein requires the free radicals to be mobile in a solution. The free radicals in the polyurethane implant are associated with the condensed aromatic structures that are stable in polyurethane and cannot propagate in the solution. When the protein is bonded with the surface, the protein does not migrate into the solution. Damaging of the protein or decay of the protein activity were also not observed. Therefore, the free radicals on the polyurethane surface do not influence the organism’s tissues. 

The endothelialisation of the implant surface is one of the important factors in biocompatibility [50]. Endothelialisation is considered a method of reducing the immune response [51]. The attachment of an active protein can significantly improve cell adhesion [52]. Fast and total endothelialisation in an organism is the primary aim for the biocompatibility of implants.

In the case of the newly synthesised polyurethane, the endothelial cells are spread out and proliferate on the surface without PIII treatment. The endothelial cells also grow well on the PIII-treated polyurethane surface. However, the untreated polyurethane is observed totally covered with endothelial cells after only 6 days, while the PIII-treated polyurethane is totally covered after 2 days, as observed on the control tissue culture plastic (TCP) [53]. 

Similar cell attachment and proliferation were observed for other ion beam and PIII-treated polymers. For example, the ion beam treatment of polystyrene by Na and Ne ions of 150 keV energy improved endothelial cell spreading, attachment, proliferation and resistance to detaching with trypsin [54]. However, this research did not explain the mechanism by which this occurred. The adhesion and proliferation of endothelial cells and astrocytes on polyether sulphone and polyurethane by ion beams due to carbonisation of the polymer surface were reported in [55]. Endothelial cell adhesion and proliferation on PIII-treated polyurethane were reported in [56]. The mechanism of improved cell adhesion via protein attachment due to free radical reactions was proposed in [10]. Thus, the attachment of a protein with biologically active conformation leads to faster endothelialisation of the PIII-treated polyurethane implant. 

The observed improvement of the polyurethane implant with PIII treatment can be considered for future soft tissue implants, such as breast implants, testicular implants, cardiovascular implants, vascular grafts, diaphragms, finger joints, ear implants, nose implants and others. The mechanism for reducing the immune response based on free radical reactions is critical to successful implantation. 

First, the free radical concentration is reduced with time after PIII treatment. Therefore, the best result for implantation is expected when the implant is treated by plasma and installed immediately. Therefore, implants should be treated in the operating theatre just before surgery. 

Second, the free radicals are sensitive to any surface contamination after the PIII treatment. Specific environmental conditions and limited contact with any other materials and devices are required before implantation into an organism. 

Third, the sensitivity of free radicals limits the sterilisation methods. Sterilisation by chemicals or gases reduces the concentration of free radicals and, consequently, the success of the implantation. Therefore, the sterilisation of the implant would be preferred before the PIII treatment. Taking into account that a plasma method is used for sterilisation [57], the PIII treatment can be completed in sterile environments such as an operating theatre. 

In general, the potential of implants without FBR cannot be overestimated. Cardiovascular implants, including heart valves, blood vessels, catheters, pacemakers and defibrillators, which do not generate FBR can be applied without immunosuppressants. Patients would not be at risk of infection after implantation. A category of patients, who are at risk of corresponding diseases and for whom an implantation has not been recommended, will be able to be treated with implants. The risk of inflammation and thrombosis can be significantly reduced. The breasts, lips and other cosmetic implants would not cause contractures. Implants with electrical connections to tissue such as cochlear, spine neurostimulator, implantable brain stimulator, different sensors in organs and the blood would never lose electrical conductivity through the fibrotic capsule in an organism. Implantable drug release devices would never lose their drug penetration rate through the fibrotic capsule. Only permanent insulin-releasing devices will save millions of patients with diabetes. Consequently, these improvements affect some millions of patients and can significantly influence their health, medical treatment and the medical industry. 

## 5. Conclusions

The investigations of organism responses to the untreated and PIII-treated polyurethane implants showed that all implants did not cause physiological dysfunctions in the mice. The PIII-treated implants in the mice showed a weaker immune response of the organism—in particular:

1. The capsule around the PIII-treated implant was significantly thinner (*p* < 0.01).
2. The macrophage activity near the PIII-treated implants was significantly weaker (*p* < 0.001).
3. The cell proliferation activity near the PIII-treated implants was significantly weaker (*p* < 0.001).
4. The proinflammatory activity by the vWF test was much weaker near the PIII-treated implants (*p* < 0.001).

Therefore, the changes in the organism responses to the PIII-treated implants in comparison to the untreated polyurethane implants were statistically significant. 

It was found that, in some cases of the surface-treated polyurethane, the immune response was neglectable. The reason for the low immune response was a covalent protein attachment on the implant surface. The protein attachment was provided by a chemical reaction of the protein molecule with a carbon atom with an unpaired electron (free radical) on the edges of condensed aromatic structures with a π-electron-stabilising cloud. The same unpaired electrons at the carbon atom on the surface were responsible for the hydrophilic interaction with the attached protein, saving the protein conformation and activity. 

Such implants with weak or without foreign body reactions can potentially be applied without suppressing the immune system. We are looking for collaborators and funding to continue these studies.

## Figures and Tables

**Table 1:** Thickness (μm) of the capsule near the polyurethane implants dependent on the PIII treatment time.

	Untreated	40 s	80 s	200 s	400 s	800 s
Thickness, μm	102 ± 21	46 ± 23	42 ± 22	38 ± 14	40 ± 6	37 ± 11

**Table 2:** Macrophage activity near the polyurethane implants dependent on the PIII treatment time.

	Untreated	80 s	200 s	400 s	800 s
Area, %	3.6 ± 1.0	1.6 ± 0.6	0.6 ± 0.3	1.3 ± 0.4	4.4 ± 1.1
Density, a.u.	9.8 ± 2.7	4.0 ± 1.4	1.7 ± 0.8	3.4 ± 1.1	13.7 ± 4.0

**Table 3:** Cell proliferation activity near the polyurethane implants dependent on the PIII treatment time.

	Untreated	40 s	80 s	200 s	400 s	800 s
Area, %	1.12 ± 0.37	0.88 ± 0.26	0.51 ± 0.30	0.47 ± 0.25	0.32 ± 0.11	0.37 ± 0.23
Density, a.u.	4.4 ± 1.5	3.3 ± 0.7	1.9 ± 1.3	1.9 ± 0.9	1.2 ± 0.4	1.4 ± 0.8

**Table 4:** Inflammatory activity near the polyurethane implants dependent on PIII treatment time.

	Untreated	40 s	80 s	200 s	400 s	800 s
Area, %	0.93 ± 0.12	0.79 ± 0.06	0.24 ± 0.10	0.35 ± 0.14	0.27 ± 0.11	0.27 ± 0.11

## Data Availability

http://hdl.handle.net/2123/20585 (accessed on 10 August 2023).
